# Is *Anopheles gambiae* attraction to floral and human skin-based odours and their combination modulated by previous blood meal experience?

**DOI:** 10.1186/s12936-020-03395-2

**Published:** 2020-09-01

**Authors:** Elison E. Kemibala, Agenor Mafra-Neto, Jesse Saroli, Rodrigo Silva, Anitha Philbert, Kija Ng’habi, Woodbridge A. Foster, Teun Dekker, Leonard E. G. Mboera

**Affiliations:** 1 Ministry of Health, Community Development, Gender, Elderly and Children, Vector Control Training Centre, P.O. Box 136, Muheza, Tanzania; 2 ISCA Technologies, 1230, West Spring St, Riverside, CA 92507 USA; 3 University of Dar es Salaam, Dar es Salaam, Tanzania; 4 Swedish University of Agricultural Sciences, Alnarp, Uppsala, Sweden; 5 BioInnovate AB, Lund, Sweden; 6 SACIDS Foundation for One Health, Sokoine University of Agriculture, Morogoro, Tanzania; 7 Department of Evolution, Ecology and Organismal Biology, The Ohio State University, Columbus, OH USA; 8 Department of Entomology, The Ohio State University, Columbus, OH USA

**Keywords:** *Anopheles gambiae*, Mosquito, Attraction, Vectrax, Skin Lure, Volatiles

## Abstract

**Background:**

Mosquitoes use odours to find energy resources, blood hosts and oviposition sites. While these odour sources are normally spatio-temporally segregated in a mosquito’s life history, here this study explored to what extent a combination of flower- and human-mimicking synthetic volatiles would attract the malaria vector *Anopheles gambiae* sensu stricto (*s.s*.)

**Methods:**

In the laboratory and in large (80 m^2^) outdoor cages in Tanzania, nulliparous and parous *A. gambiae s.s.* were offered choices between a blend of human skin volatiles (Skin Lure), a blend of floral volatiles (Vectrax), or a combination thereof. The blends consisted of odours that induce distinct, non-overlapping activation patterns in the olfactory circuitry, in sensory neurons expressing olfactory receptors (ORs) and ionotropic receptors (IRs), respectively. Catches were compared between treatments.

**Results:**

In the laboratory nulliparous and parous mosquitoes preferred skin odours and combinations thereof over floral odours. However, in semi-field settings nulliparous were significantly more caught with floral odours, whereas no differences were observed for parous females. Combining floral and human volatiles did not augment attractiveness.

**Conclusions:**

Nulliparous and parous *A. gambiae s.s.* are attracted to combinations of odours derived from spatio-temporally segregated resources in mosquito life-history (floral and human volatiles). This is favourable as mosquito populations are comprised of individuals whose nutritional and developmental state steer them to diverging odours sources, baits that attract irrespective of mosquito status could enhance overall effectiveness and use in monitoring and control. However, combinations of floral and skin odours did not augment attraction in semi-field settings, in spite of the fact that these blends activate distinct sets of sensory neurons. Instead, mosquito preference appeared to be modulated by blood meal experience from floral to a more generic attraction to odour blends. Results are discussed both from an odour coding, as well as from an application perspective.

## Background

There has been a decline of malaria incidence and prevalence globally over the past decade [1–5]. Several interventions have been reported to play substantial roles in this decline, including improved case management and malaria diagnostic methods, as well as the deployment of long-lasting insecticide-treated nets (LLINs) and indoor residual spraying (IRS) to reduce interactions between mosquito vectors and humans [2, 6–8]. However, the positive results achieved through the universal coverage of LLINs and IRS have caused the community, policy makers and other malaria control stakeholders to redirect resources toward these interventions and away from other vector control techniques [9]. Vector control campaigns, particularly those seeking to reduce transmission of malaria, have shown that over-reliance on a single approach or a certain group of insecticides leaves the campaign vulnerable due to the development of insecticide resistance [10].

Repeated application of insecticides with a similar mode of action can lead to resistance development in mosquitoes [10]. In the first global malaria eradication campaign between 1955 and 1969 intensive IRS of dichlorodiphenyltrichloroethane (DDT), although initially very successful in pushing back malaria, eventually led to DDT resistant mosquitoes and failure of the campaign in different areas [9, 11–13]. More recently, mosquito resistance to pyrethroid-based insecticides used for malaria vector control has been reported in several countries [9, 14–16]. In the face of increasing mosquito resistance and a threat of malaria resurgence, the World Health Organization (WHO) recommends the use of integrated vector management (IVM) [17], which employs several scientifically proven complementary methods of intervention to control all vectors [17]. Hence, to complement current interventions and to sustain the gains of universal coverage by LLINs and IRS, further exploration of novel and innovative strategies is of paramount importance.

LLINs capitalize on mosquitoes being attracted to humans, but intercept and kill mosquitoes before they reach the host and IRS relay on the resting behaviour of the vector. A complementary approach would be the use of odour sources other than humans to divert mosquitoes away from biting humans. These odours can be derived from flowers and extrafloral nectaries [18, 19], from oviposition sites [20], or from blood hosts, such as humans [21, 22], and can be used in, for instance, interfering with mating or oviposition or in eliminating vectors. The latter, called attract-and-kill approach, involves attracting mosquitoes to odour baits laced with a toxic agent that kills the vector upon contact. By fine-tuning blends, baits can be developed that selectively target mosquitoes and have minimal impact on the environment. These have the potential to make a significant contribution to mosquito population management and the suppression of mosquito-borne diseases [21, 23–27]. Such methods are increasingly being employed against pest insects in agriculture and in vector control.

The attraction of mosquitoes to single compounds emanating from humans has been demonstrated several times [28–30], and a single plant-based compound also has been reported to be effective [31]. In addition, studies have explored possible additive or synergistic effects of blends of semiochemicals to increase mosquito attraction, either through mimicking human volatiles [27, 32–34] or putative host plants [35]. However, initial tests of combined human and plant volatiles have been investigated only quite recently [36–39], with mixed or inconclusive results. The objective of the present study was to evaluate how combining odour blends would reduce or increase attractiveness to the major African malaria vector, *Anopheles gambiae* sensu stricto (s.s.). The rationale behind the choice of synthetic blends used was that these are characteristic for spatiotemporally distinct sources (vertebrate and floral). In addition, the blends induce sensory responses in distinct sub-sets of sensory neurons that either express ionotropic receptors (IRs), in grooved peg neurons or neurons that express olfactory receptor, and could thus behaviourally complement (addition or synergy) or antagonize (reduced attraction) each other.

## Methods

### Study area

This study was carried out in Muheza District located in the northeast of Tanzania (5°13′ S, 38°39′ E; altitude 193 m). The district is characterized by a humid and warm climate almost throughout the year. The average annual rainfall in Muheza is 1000 mm with two seasonal peaks i.e., a main peak between March and May, and a less pronounced one between November and December. The mean temperature in the area is 26 °C, with below-average temperatures between June and September and above-average between October and May. The experiments were carried out in an insectary and in mosquito spheres [40] at the Amani Research Centre of the National Institute for Medical Research.

### Rearing mosquitoes for experiments

*Anopheles gambiae s.s.* Kisumu strain, from the Kenya Medical Research Institute (KEMRI) has been maintained in a controlled environment at 27 ± 1 °C, 65 ± 5% relative humidity (RH) and a 12:12 h light–dark cycle at the Amani Research Centre since early 1982. Larvae were reared in plastic trays (20 × 30 × 10 cm) holding 500 ml of distilled water in groups of 250 per tray and fed on fish food (Tetramin^R^) once a day. Adults were kept in cages (30 × 30 × 30 cm) with access to a 10% aqueous sucrose solution for sustenance. To enable reproduction, female mosquitoes were blood-fed on rabbits according to Standard Operating Procedures (SOPs) approved by the Tanzania Medical Research Coordinating Committee [41]. European Union (EU) guidelines and standards were followed in rabbit maintenance [41]. Only female mosquitoes were used for both laboratory and semi-field trials.

### Chemical/odour blends used

Vectrax (ISCA Technologies, Riverside, USA) is sprayable liquid formulation comprised of a synthetic mix of typical floral volatiles that mimic sugar-rich flowers and extrafloral nectaries from which mosquitoes of all species and both sexes seek sustenance throughout their lives [23]. Mosquitoes detect these floral attractants, released over time from the Vectrax formulation, and respond by orienting their flight toward the point source. Vectrax also contains several sugars- and protein-based feeding stimulants, which encourage mosquitoes to feed upon the formulation to full engorgement [23]. Skin Lure is a matrix material containing human skin mimic compounds consisting of a proprietary blend of acids and ammonia and formulated in SPLAT (Specialized Pheromone and Lure Application Technology, a material that allow slow release of odour). The product was produced at ISCA technologies (Riverside, USA) and supplied in bubble caps form. The combination in this study was the combined presentation of Vectrax and Skin Lure.

### Laboratory experiments

**Fig. 1:**
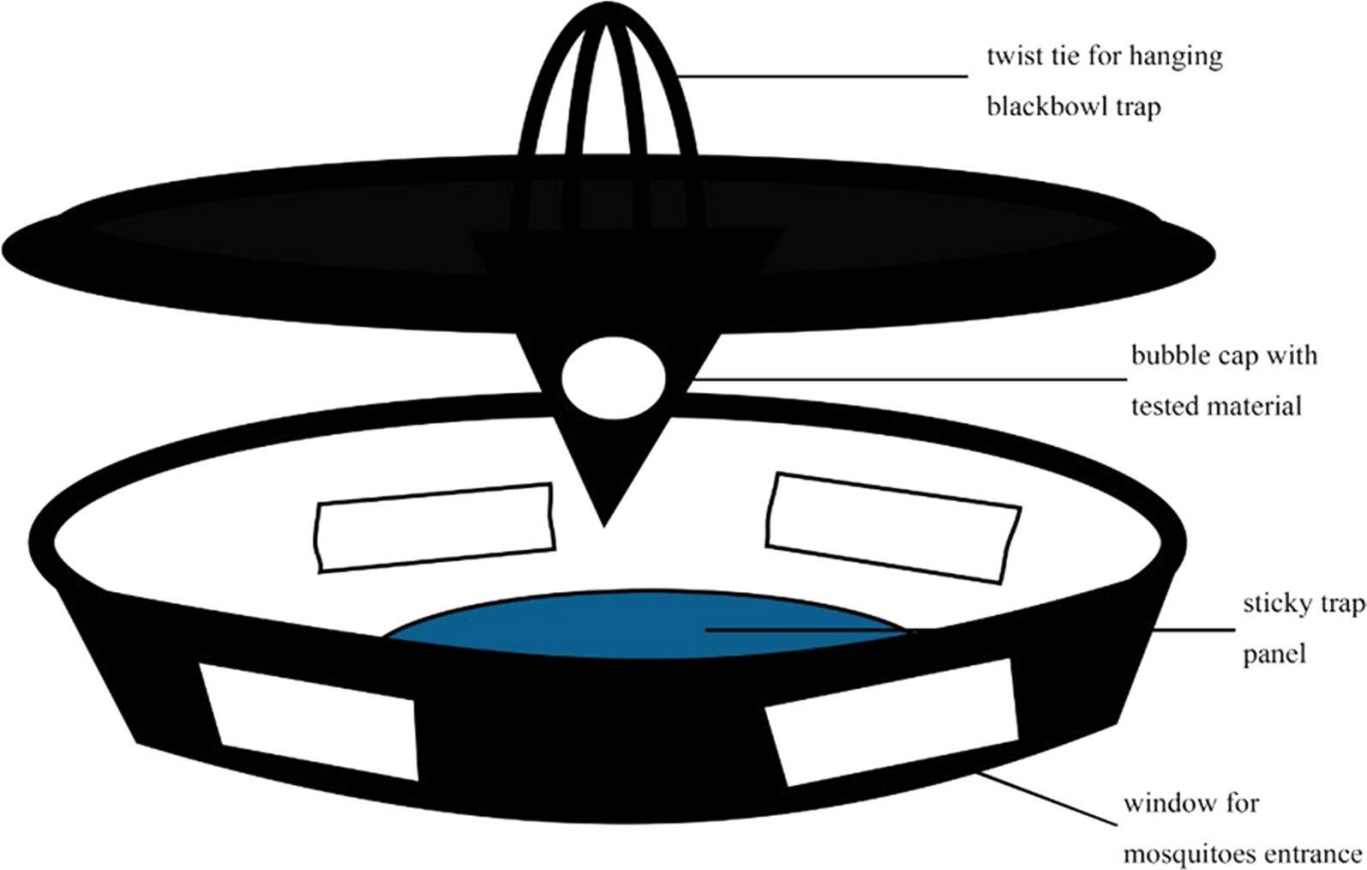
Schematic drawing of black plastic bowl trap

**Fig. 2:**
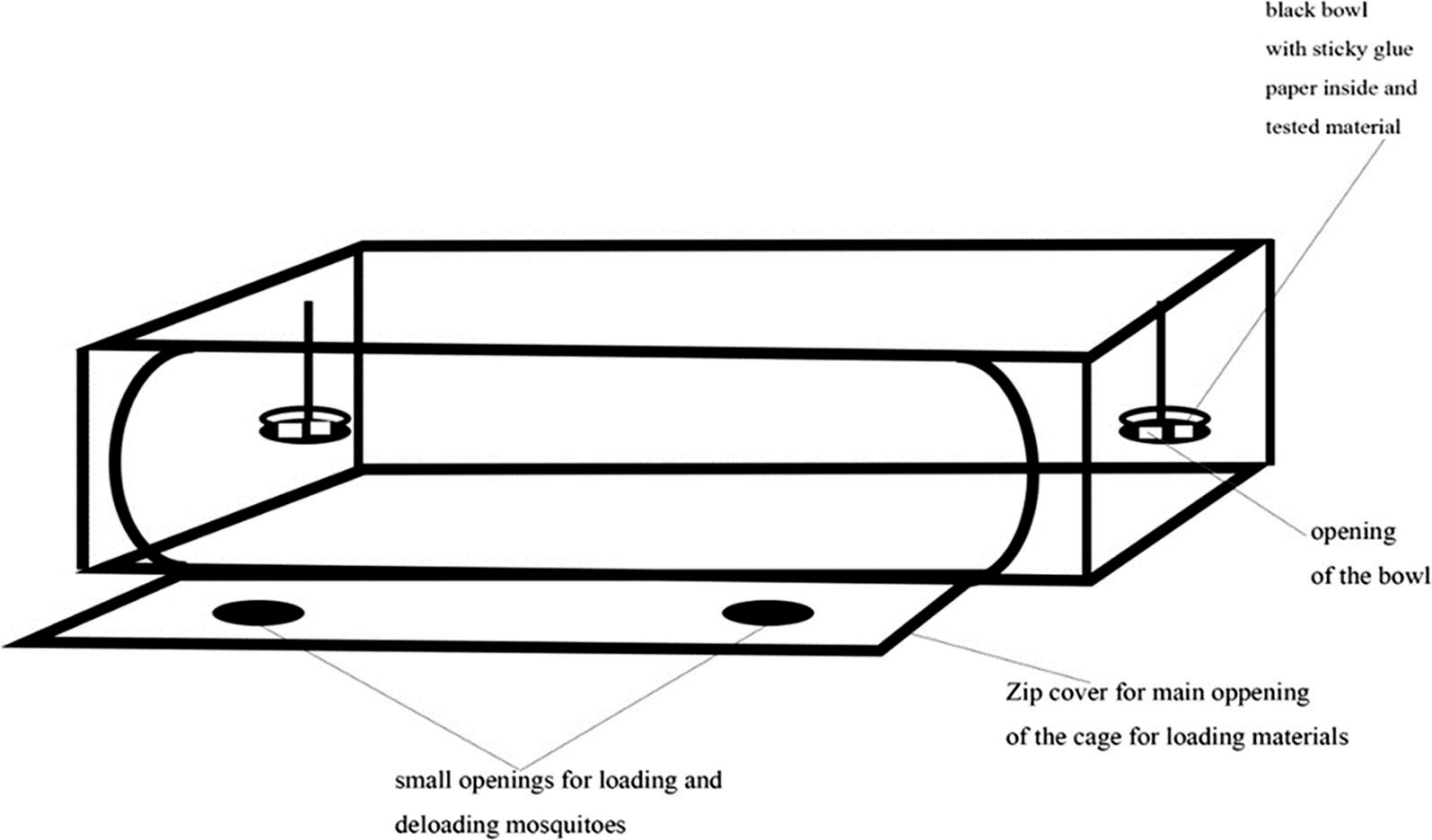
Scheme of attractants testing setup in cage trial under laboratory settings

The laboratory experiments were conducted in the insectary held at 27 ± 1 °C, 65 ± 5% relative humidity under a 12:12 h light–dark cycle. Adult females 4–5 days old were released into rectangular 91 × 46 × 30 cm mesh cages, 20 mosquitoes per cage, in which tested attractants choices were offered. In the first experiment, the attractiveness of Vectrax and Skin Lure were compared to each other with blood-fed and unfed females. In the second experiment, each attractant, Vectrax and Skin Lure, was compared against the combination in separate cages on parous and non-parous females. Each attractant was offered in a 10 × 5 × 4 cm black plastic bowl with a lid. The bowl had four evenly spaced open windows (2 x 5 cm) on the walls and a fifth one on the lid. The windows allowed mosquito access to the inside of the bowl. A small piece of green panel sticky trap with standard wet entomological glue (ISCA Technologies, Riverside, USA) was fitted onto the bottom of the black plastic bowl to trap mosquitoes that entered (Fig. 1). A petri dish with 5 ml of Vectrax was placed on the sticky-trap panel. The Skin Lure in bubble cap was hung from the window on the lid of the bowl. After one or both attractants were placed in the bowl traps, the traps containing the attractants to be compared were placed on opposing corners of each mesh screen, about 61 cm apart (Fig. 2).

Adult females, deprived of sugar for two hours before the experiment were released into the mesh screen cages 30 min prior to placing traps in the cage, to allow for acclimation. After 24 h, the number of mosquitoes caught on the sticky panel of each bowl trap was counted and recorded. Four replicate trials each, with parous and non-parous females’ mosquitoes, were performed in each experiment.

### Semi-field experiments

**Fig. 3:**
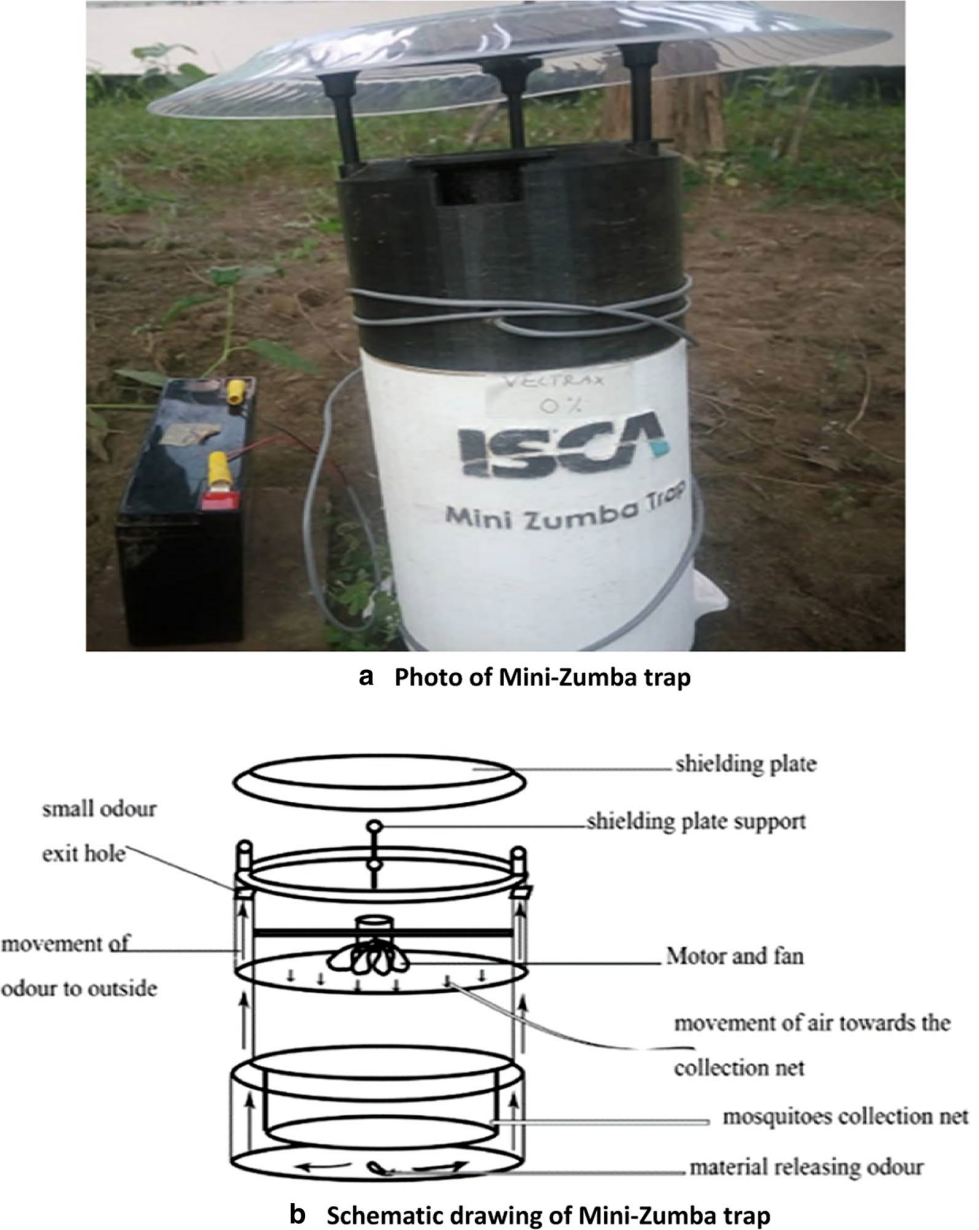
**a** Photo of Mini-Zumba trap. **b** Schematic drawing of Mini-Zumba trap

These experiments were conducted in mosquito-spheres, a greenhouse-enclosed simulation of a natural *A. gambiae* ecosystem 11.4 m length × 7.1 m width x 4.4 m height from the centre [41] over a period of 30 days. Under these settings, parous or non-parous female mosquitoes, were simultaneously offered four treatments: Skin Lure, Vectrax, a combination of the two, and a control (no attractant). In this semi-field setting, each treatment was presented using a Mini-Zumba trap (BioGents, Regensburg, Germany). Mini-Zumba traps use a fan to draw in mosquitoes from the opening on the top of the trap into a catch bag inside. The lure is placed in the bottom of the trap, outside the bag, and air that is drawn in by the fan, passes over the lure and disperses the odour. The scented air then travels up the walls of the trap and is ejected horizontally out of the baffles (holes) on the sides of the lid. This ensures that odour of the lure gets disperses away from the trap that draws the mosquitoes in Figs. 3a, b.

**Fig. 4:**
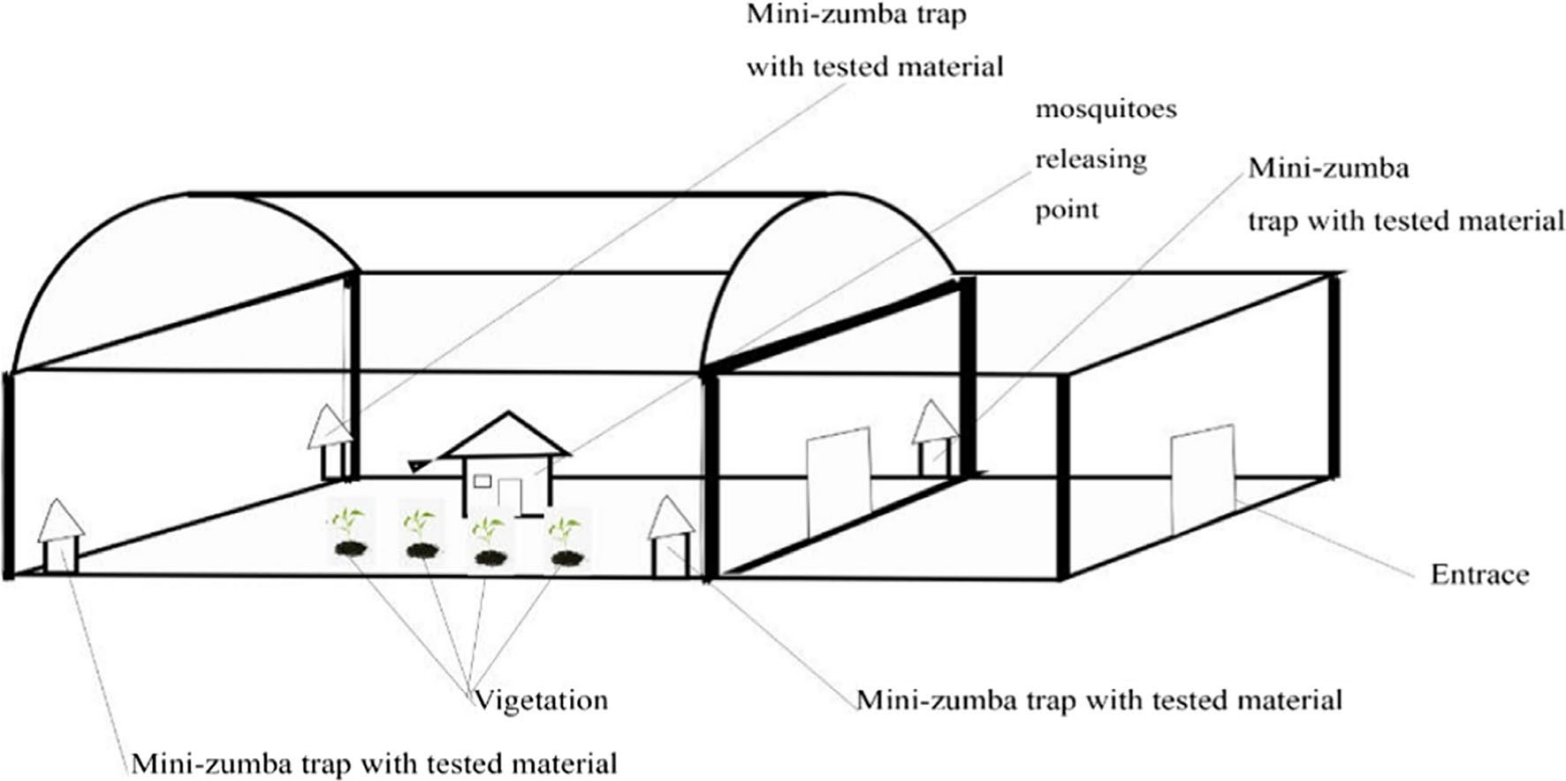
Scheme of an outdoor (semi-field) attractants trial in the mosquitoes’ sphere

The four Mini-Zumba traps were placed in each of the four corners of the mosquito sphere, approximately 6 m from each other (Fig. 4). In each experiment, a total of 200 4–5-days old adult female mosquitoes (starved for 2 h) were released in the mosquito sphere at 18:00 h. The traps were retrieved the following morning at 06:00 h. Mosquitoes captured in the collection net of each trap were collected, counted, and recorded. Mosquito collection proceeded for 2 days consecutively with no new mosquitoes being released. A buffer of 1 day was maintained for cleaning and allowing the sphere to get aired out, trapping net and attractants holding containers of the traps were emptied, and whole mini-Zumba traps cleaned. Also, this allowed uncaptured mosquitoes to die before the next experiment. A total of 2000 females (parous = 1000; non-parous = 1000) were released. The experiments were replicated five times each for both parous and non-parous mosquito experiments. In each experimental replicate, attractants were shifted position to correct for positional bias.

### Data analysis

The number of mosquitoes captured by the presented attractant in each treatment was summarized in Microsoft Excel. Choice data were general linear model fitted with a binomial distribution (GLM), followed by a pair-wise comparison using multiple comparison [42] Analysis was performed in R (version 3.4.4, R Core Team 2018). Plots were constructed in Microsoft Excel (laboratory results) and using ggplot2 in R for semi-field results [43].

### Ethical considerations

The study was approved by the Medical Research Coordinating Committee of the National Institute for Medical Research, Tanzania (Research Permit Ref. No. NIMR/HQ/R.8a/Vol.IX/1584).

## Results

### Laboratory experiments

**Fig. 5:**
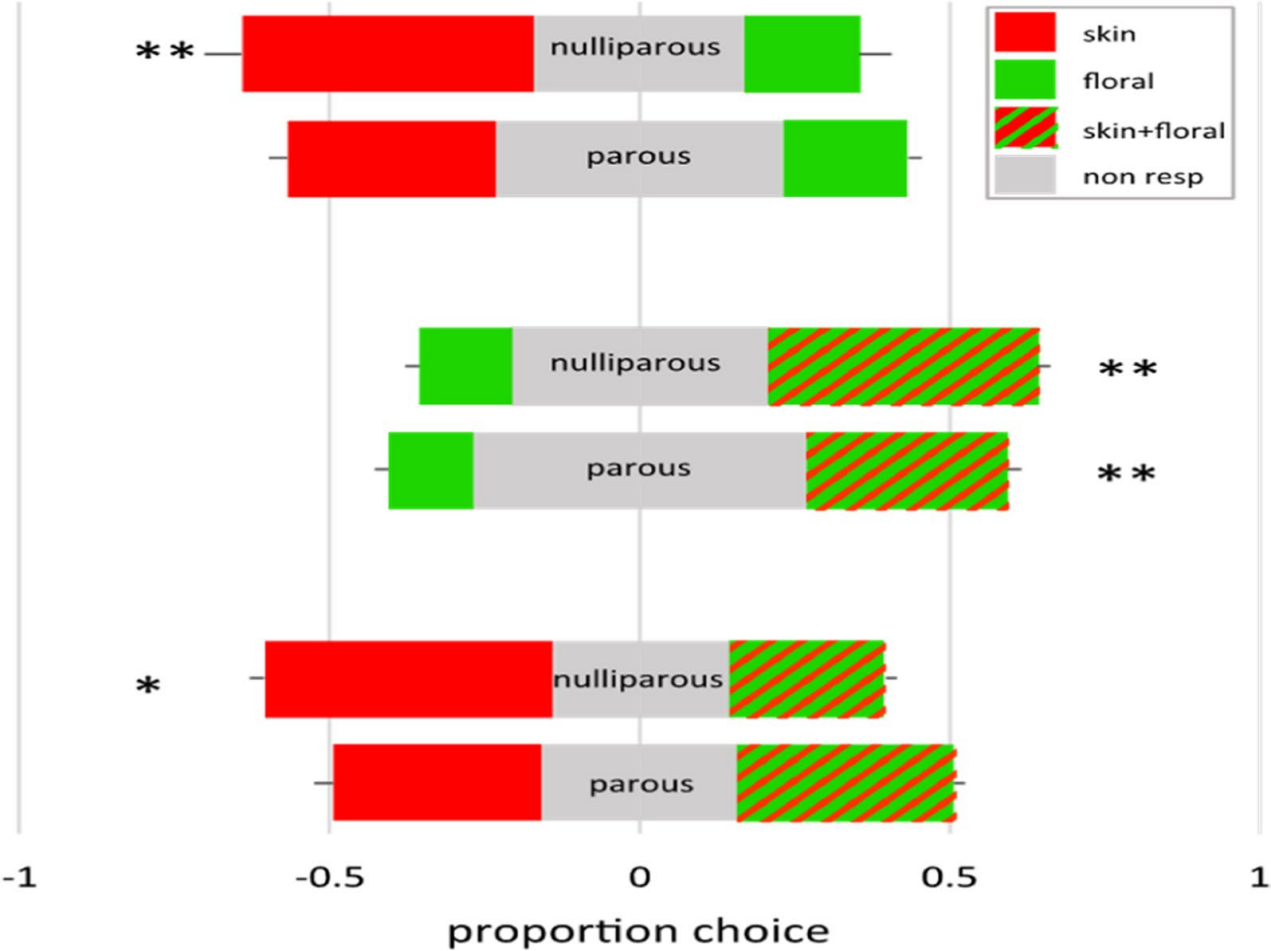
Two-choice tests in the laboratory with nulliparous and parous mosquitoes using Skin Lure, Vectrax, and their combination. Numbers were expressed as proportions and averaged over four trials. Each bar represents 100%, with in grey the proportion of traps. Error bars depict the standard error. * p < 0.05, **p < 0.01

In the laboratory experiment, a total of 292 females were captured in the traps, 157 non-parous and 135 parous out of 480 female mosquitoes that were released during the experiment. Floral odours caught significantly fewer than a combination of floral + skin odour (P 0.001), or skin odour alone (not significant for nulliparous females). Nulliparous females slightly preferred skin odour over a combination floral + skin odour. However, choices between nulliparous and parous females did not differ (Fig. 5).

### Semi-field experiments

**Fig. 6:**
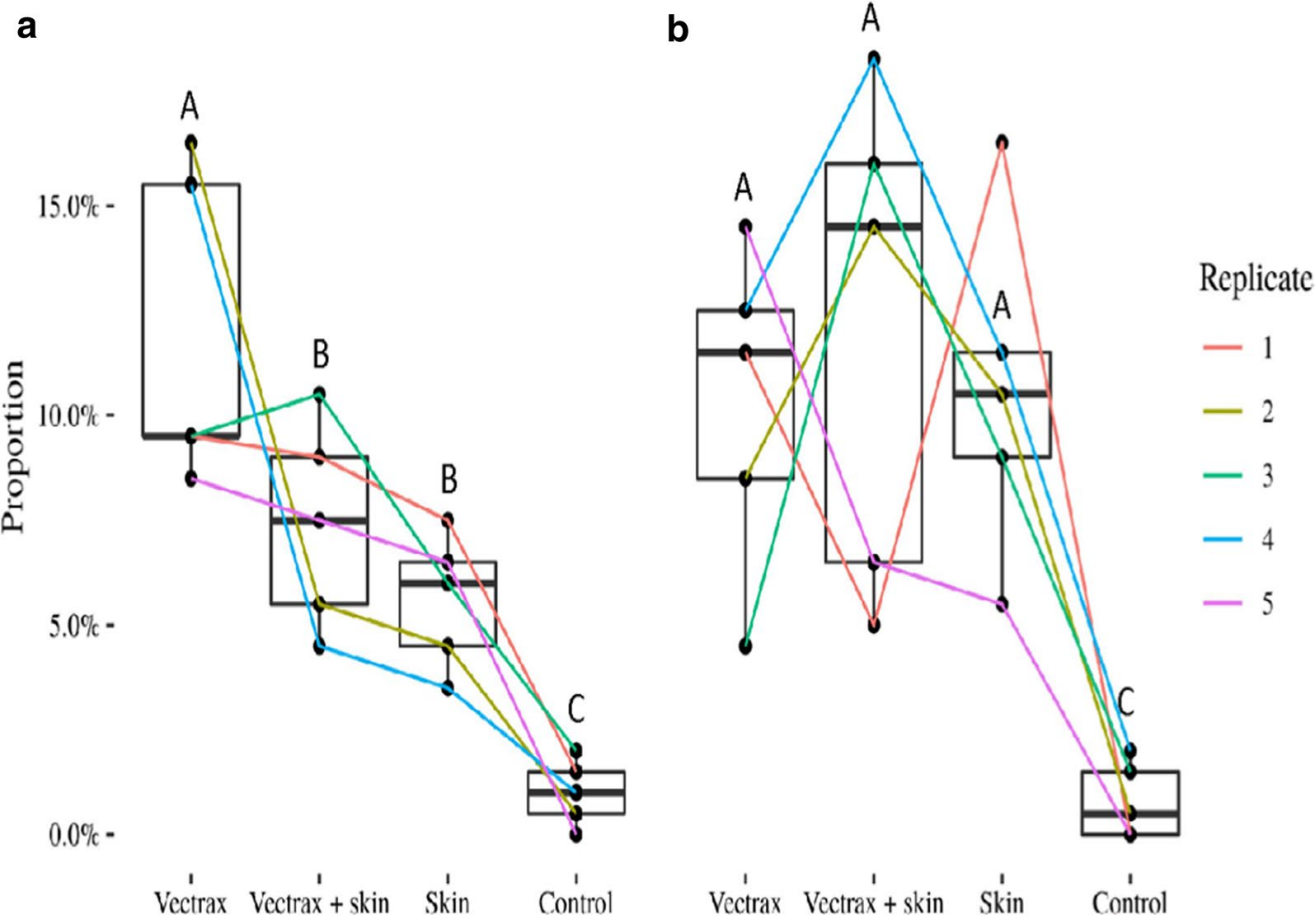
Proportion recapture of nulliparous (**a**) and parous (**b**) mosquitoes in mosquito spheres (cages set up in the field) in which they were offered four choices, including floral odour (Vectrax), skin or (Skin odour), their combination and unbaited traps. Results as depicted as box plots with the median and 75% percentiles. N = 5 with lines connecting proportions between baits in each replicate. Different letters above the box plots indicate significant differences, both within each factor (nulliparous/parous) as between them

In the mosquito-sphere trial recapture rate of nulliparous mosquitoes was significantly lower than that of parous mosquitoes (25.9 and 33.8%, p < 0.05). Traps baited with floral odour caught significantly more nulliparous mosquitoes then either skin odour or a combination of floral odour with skin odour (Fig. 2, p < 0.05,). In contrast, parous mosquitoes were equally captured by the two lures and their combination. The control treatment caught significantly fewer nulliparous and parous mosquitoes (p < 0.0001) (Fig. 6).

## Discussion

Odour-based mosquito control tools slowly find their way into application, thereby diversifying the toolbox available to local vector control schemes. With much of mosquito life revolving around odours, methods that harness a mosquito sense of smell hold great promise in providing novel tools. A broad array of sensory neurons ‘hardwire’ mosquito preference and tune its nose to resources important for survival and reproduction. A mosquito’s needs, however, frequently change between mating, nectar feeding, blood feeding and oviposition, and with that the odours to which it orients. Mosquitoes thus have to ‘toggle’ between sensory modes, which involves peripheral [44, 45] and/or central modulatory factors [45]. In this study, we evaluated whether a combination of odours from spatio-temporally different origins would synergize attraction, or alternatively, constitute olfactory nonsense to a mosquito nose and potentially mask attractiveness. Parous mosquitoes switch in behaviour from nectar feeding to a combination of blood host and nectar feeding [19]. Comparison between parous and non-parous female mosquitoes may thus highlight differences that are due to this switch in preference. This study showed that combining synthetic mimics of floral and human odour attract nulliparous and parous mosquitoes. As field populations are comprised of mosquitoes whose odour preferences vary with, for example, age, nutritional and gonotrophic state, such complex, multiplexed blends may be more effective and take a broader sweep of the mosquito population.

Over the past 60 years, attraction of female mosquitoes to blood-host mimicking odours and plant-based attractants has rarely combined odours from different origin [46–53]. In those studies, where odours of presumed floral and vertebrate origin were combined, mixed results were obtained, by and large not indicating synergy [36–39]. In this study, although mosquitoes were attracted to a combination of floral and human odour, the combination did not augment or synergize capture rates, in spite of each blend individually being attractive. This is largely in line with earlier reports [36–39]. In a recent field studies in Kenya, a combination of plant- and human-derived odours was observed to elicit a masking effect in trapping *Aedes aegypti* [38].

Somehow the added sensory input does not translate in an enhanced ‘attractiveness’ of the signal. This could in part be because the nutritional status of the mosquitoes in this study cohorts was similar, whereas ‘synergy’ or ‘augmentation’ of trap capture for a multiplexed lure would more readily emerge in field populations with mosquitoes in diverse physiological states. Further, it may also be that odour sources, although placed in very close proximity of each other, do not create fully merged plumes, which mosquitoes may perceive as two separate sources instead of an augmented single source. Indeed, insects are exquisitely capable of neurologically parse incompletely mixed strands of odours [54, 55].

Of further interest is the observation that in semi-field experiments nulliparous females preferred floral volatiles to other blends, whereas this preference disappeared in parous females. This demonstrated a well-known mosquito food proclivity. Female mosquitoes generally take sugar meals before they seek a blood meal, and some species strongly prefer sugar over blood or rarely bite until after a sugar meal or even not until after several weeks of sugar feeding [19, 56]. The relatively young (4–5 days old) and nulliparous females in this study may thus follow such pattern and first cater to their low energy reserves before seeking blood. In contrast, parous females, which likely have increased their energy levels through a previous blood meal were equally attracted to either lure, as they are known to alternate between sugar meals and blood meals [19].

Combining the floral and skin odour blends is also of interest as they induce sensory activity in entirely different classes of sensory neurons, with floral odours being detected by olfactory receptors (ORs), whereas the detection of the human odour blend, consisting of amines and acids, is entirely restricted to ionotropic receptors (IRs) expressed in grooved peg sensilla [57]. Accordingly, the input from floral and human odour is complementary and induces responses in separate olfactory sub-circuits [58]. Combination of input from these sub-circuits often leads to synergistic trap catches in other insect taxa example in *Drosophila* flies [59]. In mosquitoes, however, the relative importance of the OR and IR sub-circuitry may differ between distinct behaviours, such as orientation to nectar *versus* blood host resources [55]. How a combination of input from these classes of sensory neurons influences capture rates in mosquitoes, and for example, synergize capture rates of each blend separately, has not been systematically analysed. The tests performed here indicate that different from some other insect taxa, IR and OR input does, perhaps surprisingly, not necessarily synergize. Whereas this may indicate a fundamental odour-coding difference between the taxa, it may also simply be due to that the combination, release rates and ratios require further adjustment. Inconsistency between laboratory and semi-field results observed is likely to be due to the fact that in the laboratory there was closeness of the mosquitoes, hence relatively sensing high concentration while in the semi-field the large space and weather had dilution effect.

The results further suggest that a previous blood meal experience modulates olfactory preference. Shifts in blood host preference have been reported for mosquitoes [60, 61]. Similarly, shifts in preference have been found depending on internal state, such as age, mating status, physiological status, and blood feeding status [62–64]. The modulation observed here, from floral to skin odour, also implies that following a blood meal mosquito may increasingly ‘weigh’ input from the IR circuitry, tuned to vertebrate hosts, in behavioural preference. Further research is needed to more in-depth evaluate the protracted effects of a blood meal on nutritional status and preference modulation, as implied by results in this study.

From an applied perspective the results offer interesting angles. Although the study did not find any augmentation of trap catches by combining floral and skin odours, the combination did catch both nulliparous and parous mosquitoes (which differs slightly from earlier reports [37–40]), and would therefore attract mosquitoes relatively independently of physiological status, these being either searching to replenish carbohydrate energy reserves for flight and maintenance (floral odours) [19, 65, 66] or searching for hosts to support reproduction (skin lure, a human skin-mimicking blend of volatiles) [28, 67, 68]. Although there was significant attraction to either floral and skin odour blends, alone and in combination, to both parous and non-parous females, this study did not compare the attraction of the blends to a living human. Further research is needed to assess the attractiveness at different concentrations of the materials and new odour blends compared to that of humans in natural field settings.

## Conclusion

Multiplexing volatiles of spatio-temporally segregated odour sources can attract mosquitoes in different physiological state. Captures with such a bait may sample mosquito populations more broadly and represent mosquito populations more accurately. In addition, such lures may be used in novel attract-and-kill methods that not only attract young and nulliparous mosquitoes out for a carbohydrate source, but also parous and blood host-seeking individuals that may already be infected with malaria, and thus doubly impact mosquito longevity and malaria transmission. Fine-tuning such lures to target mosquitoes selectively can further increase efficacy, environmental friendliness and prospect in future application.

## Data Availability

All available data are included in this article.
